# Modification of Closed-State Inactivation in Voltage-Gated Sodium Channel Na_v_1.7 by Two Novel Arachnid Toxins

**DOI:** 10.3390/toxins17090432

**Published:** 2025-08-29

**Authors:** John W. Johnson, Hillary G. Rikli, Stephen R. Johnson

**Affiliations:** 1 Pharmacology Department, Southern Illinois University School of Medicine, Springfield, IL 62702, USA; jjohnson88@siumed.edu; 2 Carbon Dynamics Institute, LLC, Springfield, IL 62707, USA; hillary.rikli@gmail.com

**Keywords:** closed-state inactivation, high resolution mass spectrometry, mass-to-charge ratio, retention time, time-of-flight, total ion chromatogram, ultra-performance liquid chromatography, voltage-sensing domain

## Abstract

Venomous invertebrates have provided a large diversity of toxins that selectively and potently modulate ion channels that are indispensable tools for elucidating the structure and underlying mechanisms of these channels. Voltage-gated sodium channels (VGSC) are responsible for the initiation and propagation of action potentials in excitable cells and represent an important target for a variety of diseases. The Na_v_1.7 isoform, located in the peripheral nervous system, is central to pain signaling and is under intense investigation as a target for the treatment of pain. Closed-state inactivation (CSI) has been implicated in various disease states, such as arrhythmias and neuropathic pain. The investigation of venom toxins and VGSC CSI is poorly understood. However, many scorpion and spider toxins bind to site 3, characterized by a delay in steady-state inactivation, and interact with domain IV of the channel alpha subunit. In this study, two novel toxins were isolated from the venoms of *Heteroctenus junceus* and *Poecilotheria regalis* that demonstrated similar activity to site 3 modulators. Both toxins were shown to inhibit CSI while enhancing the rate at which CSI can occur. Taken together, this study demonstrates the need for additional investigation in CSI as well as the ability for toxins to modulate this phenomenon.

## 1. Introduction

Voltage-gated sodium channels are significant integral membrane protein targets for future pharmacological intervention. VGSC plays a crucial role in the initiation and propagation of action potentials [1]. VGSC pore-forming α-subunits are distinguished by nine isoforms, namely Na_v_1.1 through 1.9 [2]. The VGSC isoform Na_v_1.7, expressed primarily in the peripheral nervous system (PNS), plays a central role in pain signaling. This is demonstrated by Na_v_1.7 mutations that have been shown to result in either gain- or loss-of-function that modify pain signaling [3]. These Na_v_1.7 mutations have been shown to be an underlying causation of a variety of neuropathic pain disorders such as small-fiber neuropathy (SFN), inherited erythromelalgia (IEM), and paroxysmal extreme pain disorder (PEPD), as well as the loss of pain exhibited by congenital insensitivity to pain (CID) [4,5,6,7]. These mutations highlight the importance of further Na_v_1.7 investigations and demonstrate its great potential as a novel target to treat pain.

The VGSC α-subunit is made of four homologous domains (DI-IV), each consisting of six transmembrane segments (S1–S6), and is sufficient to produce action potentials [8,9]. S1–S4 form the voltage-sensing domain (VSD), and S5 and S6 form the channel pore [10]. Activation of each VSD is brought about when S4 moves outward upon cell membrane depolarization, leading to channel activation [11,12]. The phenomenon known as fast inactivation is produced by the activation and subsequent movement of DIII and DIV, which exposes an intracellular binding region for the linker between DIII and DIV, containing a conserved IFM motif across all isoforms, that upon binding terminates sodium conductance, referred to as the “hinged-lid” model [13,14,15,16,17,18,19]. Fast inactivation is the typical means of inactivation and can be recovered upon sufficient time following repolarization [20]. Slow inactivation is a second type of inactivation that is mechanistically distinct from fast inactivation. Slow inactivation requires a long or repetitive stimulus to elicit and is followed by an increased duration of inactivation and is not immediately recovered upon repolarization [20,21]. As opposed to the blockage of the channel by means of the DIII-DIV linker, the mechanisms underlying slow inactivation involve the VSD, selectivity filter, the activation gate, and the fast inactivation gate [22].

The inactivation of a VGSC is further characterized as inactivation following an open state or from a pre-open closed-state, open-state inactivation (OSI) and closed-state inactivation (CSI), respectively [23,24,25,26]. While the activation of all four VSDs is required for activation, the participation of all VSDs is not required for inactivation. The contribution of the DIII-DIV VSD activation is thought to be sufficient to allow the DIII-DIV linker to move, hindering sodium conductance and inactivating the channel before channel opening can occur [14]. CSI can be elicited from slight subthreshold depolarizations [24,25]. Preference for either OSI or CSI can vary depending on the isoform and, due to mutations, has been shown to be an underlying factor contributing to several disease states [27,28,29,30]. Further understanding of VGSC inactivation can be studied by the interaction of modulatory proteins with observable kinetics.

Venom-derived toxins offer a rich spectrum of compounds that interact with VGSC by a variety of mechanisms. Toxins often display channel selectivity as well as specific modulation [31]. This provides a unique opportunity to study various means of modulating VGSC that have exposed seven unique binding regions that interact with venom-derived toxins [32]. One of the VGSC binding regions that has been shown to modify inactivation is site 3, with toxins having been identified from sea anemones, scorpions, and spiders [33]. These toxins slow inactivation of sodium channels by preventing the conformational change in DIV to produce inactivation [34]. Site 3 toxins, such as α-scorpion toxins, experimentally produce an observed decrease in inactivation as demonstrated by the prolongation and persistence of sodium currents produced by VGSC in the presence of toxin as well as an increase in the peak sodium conductance [32,33,35,36]. Site 3 toxins have demonstrated enhanced recovery from inactivation [36,37]. They have been shown to bind to residues on the loops between S3 and S4 as well as between S5 and S6 of DIV [38,39]. Some toxins modulate site 3 but have also been shown to bind to site 4. Site 4 toxins are associated with DII and typically modify the kinetics of the channel’s activation by trapping the VSD in the activated position [40]. The α-scorpion toxin OD1, which is associated with site 3, has also been shown at higher concentrations to have characteristics of site 4 modulators, like structurally similar β-scorpion toxins [41]. Several spider toxins, such as δ-palutoxins, β/δ-agatoxins, and Poecilotheriatoxins, are now also thought to potentially bind to these two sites [42,43,44,45].

Since promiscuous activity is sometimes observed, our understanding of these modulatory sites becomes more confusing and may require better elucidation for future pharmacophore targeting. The examination of site 3 toxin’s effect on CSI is one activity that has been understudied and demonstrates potential disagreement with site 3 toxins classification. The sea anemone toxin, Anthopleurin A (AP-A), was shown not to alter CSI of Na_v_1.5 but was shown to enhance CSI of Na_v_1.4 [46,47]. Alternatively, Jingzhaotoxin-II (JZTX-II), a 32 residue peptide toxin from *Chilobrachys jingzhao*, has been shown to hinder Na_v_1.5 from entering CSI [48]. Both AP-A and JZTX-II have been shown to interact with site 3 [47,49]. To further examine this discrepancy, the effect on CSI by other toxins that exhibit modulation of inactivation in a manner like that of site 3 toxins will need to be examined. Here, we explored the venoms of *Heteroctenus junceus* and *Poecilotheria regalis* and identified two novel toxins that have similar profiles to other site 3 toxins that hinder Na_v_1.7 from entering CSI.

## 2. Results

### 2.1. Toxin Selection and Primary Structure Determination

The venom from scorpions (order Scorpiones) and spiders (order Araneae) typically has a complex mixture of low molecular weight proteins ranging from 1000 to 10,000 Da. Although they may also contain smaller amounts of larger proteins, i.e., >10 kDa, it is the smaller peptides that are under intense study for their selective and potent modulation of ion channels. In this study, venoms from representative species, i.e., *Heteroctenus junceus* and *Poecilotheria regalis*, were collected, prepared, and analyzed in parallel to explore their modulatory effects on Na_v_1.7.

**Figure 1:**
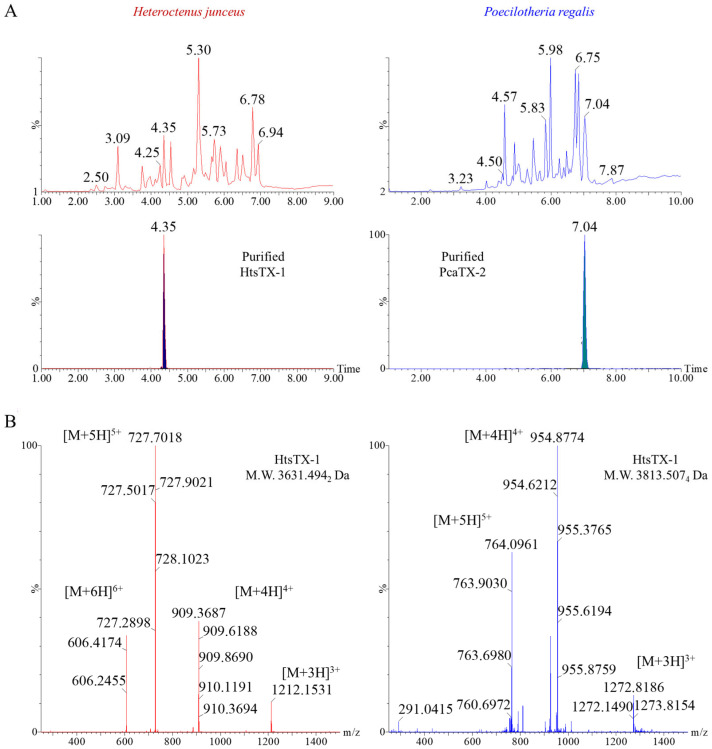
**Representative TICs of *Heteroctenus junceus* and *Poecilotheria regalis* venom.** A reversed-phase liquid chromatographic separation was performed for both *H. junceus* and *P. regalis* venom (see Methods 5.4). (**A**) A representative TIC for each whole venom (top) shows a complex mixture of small peptides and proteins. One toxin was purified from each venom (bottom) and were designated Heteroctenustoxin-1 (HtsTX-1) and Poecilotheriatoxin-2 (PcaTX-2). (**B**) A multiply charged electrospray spectrum with the [M + 5H]^5+^ and the [M + 4H]^4+^ ion clusters identified confirm that both HtsTX-1 and PcaTX-2 are compact peptides with molecular monoisotopic masses of 3631.4942 and 3813.5074 Da, respectively.

To obtain preliminary compound characteristics, raw venom from each species was freeze-dried, and 100 μg was suspended in the initial mobile phase. Each suspension was analyzed by UPLC coupled to a HRMS, providing conventional reversed-phase separation and high-resolution MS spectra. The total ion chromatogram (TIC) for venom from each species displays a myriad of low molecular weight peptides with differential characteristics and very little conservation between species (Figure 1A Top). UPLC fractions were screened for Na_v_1.7 site 3 activity, and those that tested positive were retained for further characterizations. While there were several candidates from each venom, one novel peptide was chosen from each species and selected for this investigation due to their size, Cys bridge configuration, high abundance, and resemblance to previously described site 3 modulators. The purified toxins (Figure 1A Bottom) were identified as Heteroctenustoxin-1 (HtsTX-1) and Poecilotheriatoxin-2 (PcaTX-2). Using an averaged lock mass corrected multiply charged electrospray spectrum, the monoisotopic molecular weights were determined to be 3631.4942 and 3813.5074 Da, respectively. A raw, unprocessed spectrum (0.5 s scan) is provided to demonstrate the classic charge envelope series seen in cysteine bridged peptides (Figure 1B).

**Figure 2:**
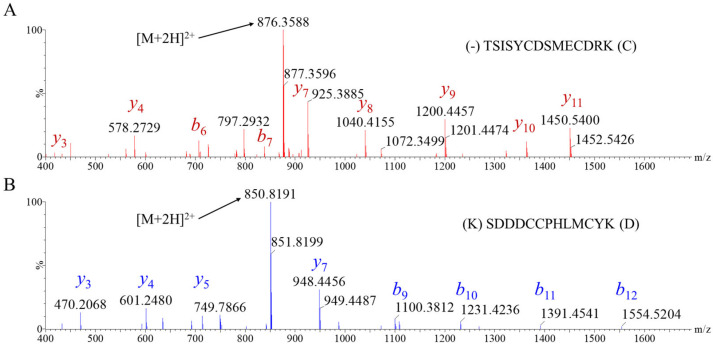
**Representative tryptic peptide fragmentation of HtsTX-1 and PcaTX-2.** A reversed-phase liquid chromatographic separation was performed for protease digested purified toxins. (**A**) A representative spectrum for tryptic peptide T1-2 of purified HtsTX-1 is provided to show the predictable Biemann *b* and *y* ion series for collision induced dissociation. (**B**) Similarly, a representative spectrum for tryptic peptide T4 of purified PcaTX-2 is provided. Both peptides were fragmented with a collision voltage ramp from 25 to 30 V. The doubly charged peptide is still visible at these energies to keep higher mass fragments intact for identification and sequencing.

To determine the primary structure of each peptide, 1 μg aliquots of purified toxins were digested with three different enzymes: trypsin, chymotrypsin, and Asp-N. Subsequently, the digests were analyzed by HRMS with both collision-induced dissociation (CID) and electron transfer dissociation (ETD) in data dependent acquisitions (Figure 2). Although no PTMs were identified in HtsTX-1, PcaTX-2 had an amidated C-terminus. This assignment of disulfide bridges was assumed to be conserved and followed the same pattern of linkages as seen in most related three-bridged scorpion and spider toxins, i.e., C1:C4, C2:C5, and C3:C6. To confirm this hypothesis, we used two analytical approaches by varying reduction, enzyme specificity, and fragmentation formats.

The primary structure of Heteroctenustoxin-1 (HtsTX-1) and Poeciolotheriatoxin-2 (PcaTX-2) were determined. HtsTX-1 is a 32-residue peptide with three disulfide bridges:

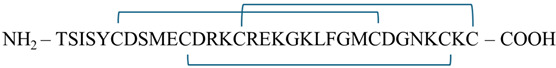



PcaTX-2 is a 33-residue peptide with three disulfide bridges:

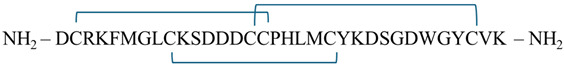



### 2.2. Effect of Toxins on Na_v_1.7 Currents

**Figure 3:**
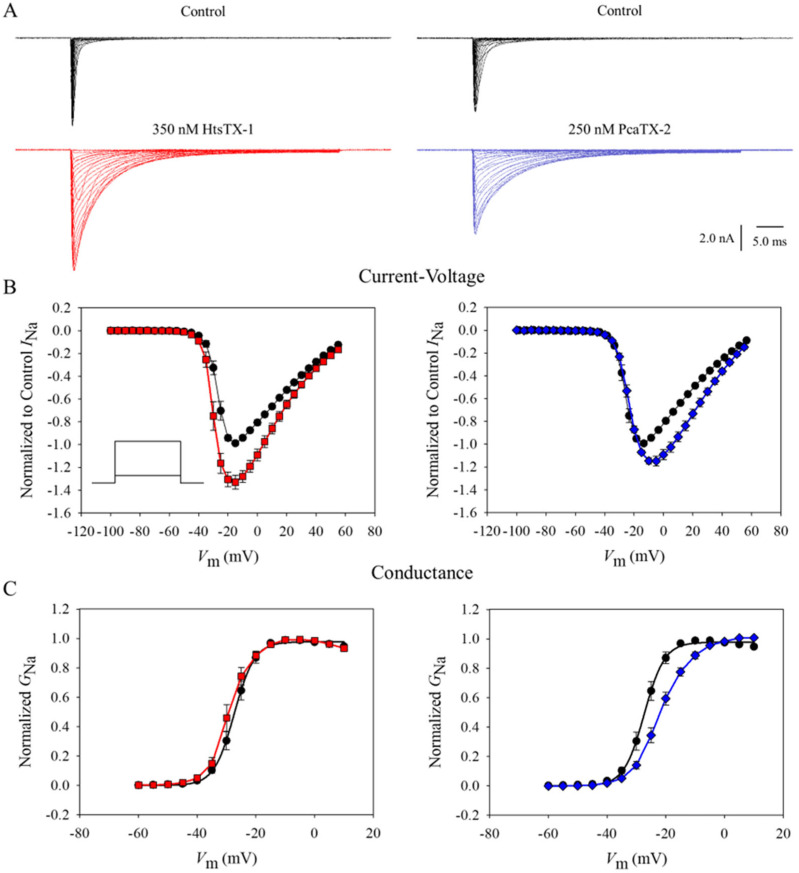
**The effect of HtsTX-1 and PcaTX-2 on Na_v_1.7 currents.** (**A**) Cells were kept at a holding potential of −120 mV and subjected to 50 ms depolarizing pulses from −100 to 60 mV in +5 mV increments in control conditions (black) and after application with 350 nM HtsTX-1 (red) and 250 nM PcaTX-2 (blue). (**B**) Normalized *I*/*V* (current–voltage) curves pre- (●) and post-application with HtsTX-1 (red ■) and PcaTX-2 (blue ♦) were produced, and *V*_½_ and *V*_rev_ were determined from the fitted curve. Neither HtsTX-1 nor PcaTX-2 demonstrated a statistically significant change in *V*_½_ or *V*_rev_. The *I*/*V* curves show an increase in the peak current caused by the HtsTx-1 and PcaTX-2 of 39.6 ± 3% and 19.8 ± 2, respectively. (**C**) Normalized *G*/*V* (conductance–voltage) curves for Na_v_1.7 currents pre- (●) and post-application of HtsTX-1 (red ■) and PcaTX-2 (blue ♦) were fitted to a Boltzmann distribution. Both HtsTX-1 and PcaTx-2 caused a small, statistically significant shift in *G V*_½_. There was no significant difference for the *G k* for HtsTX-1 treatment compared to control; however, PcaTX-2 significantly increased the *G k*.

As previously discussed, the focus of this research was to compare the site 3 modulation of venom from two representative species of class Arachnida. Due to their neuronal origin and high expression of Na_v_1.7, CAD cells were chosen to probe for this sodium channel modulation [50]. Purified HtsTX-1 and PcaTX-2 were diluted in extracellular solution at a concentration of 350 and 250 nM, respectively, immediately before analyses and used throughout these experiments. Using a perfusion flow of 0.5 mL/min, no recordings were made post drug application until a maximum and equilibrated effect was observed to ensure no bias occurred due to differences in binding. Currents were induced by 50 ms depolarizing steps in 5 mV increments from −100 mv to +60 mV from a holding potential (*V*_H_) of −120 mV. Representative current traces prior to (black) and after HtsTX-1 (red) or PcaTX-2 (blue) toxin application are provided to demonstrate their overall effect on Na_v_1.7 currents (Figure 3A). Both peptides caused a significant increase in peak sodium current after application. In addition, both peptides caused a significant slowing in the rising decay phase of the current. The time constants (τ_F_) of steady-state fast inactivation were plotted against voltage (τ/*V*) before and after treatment with HtsTX-1 and PcaTX-2. Both treatments caused a significant shift in the τ_F_ at all voltages as compared to the control. Taken together, these data suggest a site 3 interaction.

To further characterize the modulatory effects of HtsTX-1 and PcaTX-2 on the activation kinetics of Na_v_1.7, a normalized current–voltage (*I*/*V*) plot was generated and fitted (Figure 3B). Both curves showed a significant increase in peak current caused by the HtsTX-1 and PcaTX-2 of 39.6 ± 3% and 19.8 ± 2%, respectively. Our preliminary conclusions suggested the depolarizing shift in activation caused by PcaTX-2 is like that of PcaTX-1 and resulted in a small concomitant reduction in ion driving force [45]. The half-maximal voltage (*I V*_½_) was −32.42 ± 1.11 mV for the control; this did not significantly differ from −35.13 ± 1.21 mV after application of HtsTX-1 (Table 1). The half-maximal voltage (*I V*_½_) was −33.04 ± 0.95 mV for the control; this did not significantly differ from −30.43 ± 0.86 mV after application of PcaTX-2. The reversal potential (*V*_rev_) of 61.1 ± 0.76 mV for the control did not significantly differ from 59.1 ± 0.62 after application of HtsTX-1 nor did the *V*_rev_ of 57.6 ± 1.08 significantly differ from 60.7 ± 1.19 after application of PcaTX-2. This observation suggested that the toxins do not modify the channel’s selectivity for sodium ions [51]. The voltage dependence of Na_v_1.7 activation was measured by plotting conductance–voltage (*G/V*) curves, which were then fitted by a Boltzmann distribution (Figure 3C). The half-maximal activation of current (*G V*_½_) before application was −26.75 ± 0.29 mV, which showed a small but significant negative shift to −29.35 ± 0.36 mV after application with HtsTX-1. The −21.62 ± 0.30 mV after application with PcaTX-2 significantly differs from *G V*_½_ before application of −27.35 ± 0.29 mV. There was no statistical difference in the slope factors (*G k*) before and after HtsTX-1 application; however, PcaTX-2 significantly altered the slope. Taken together, these data suggest that both HtsTX-1 and PcaTX-2 produce small changes in the voltage dependence of activation by shifting to slightly more negative potentials in the case of the former and positive potentials for the latter.

### 2.3. Effect of Toxins on Na_v_1.7 Fast and Slow Inactivation

**Figure 4:**
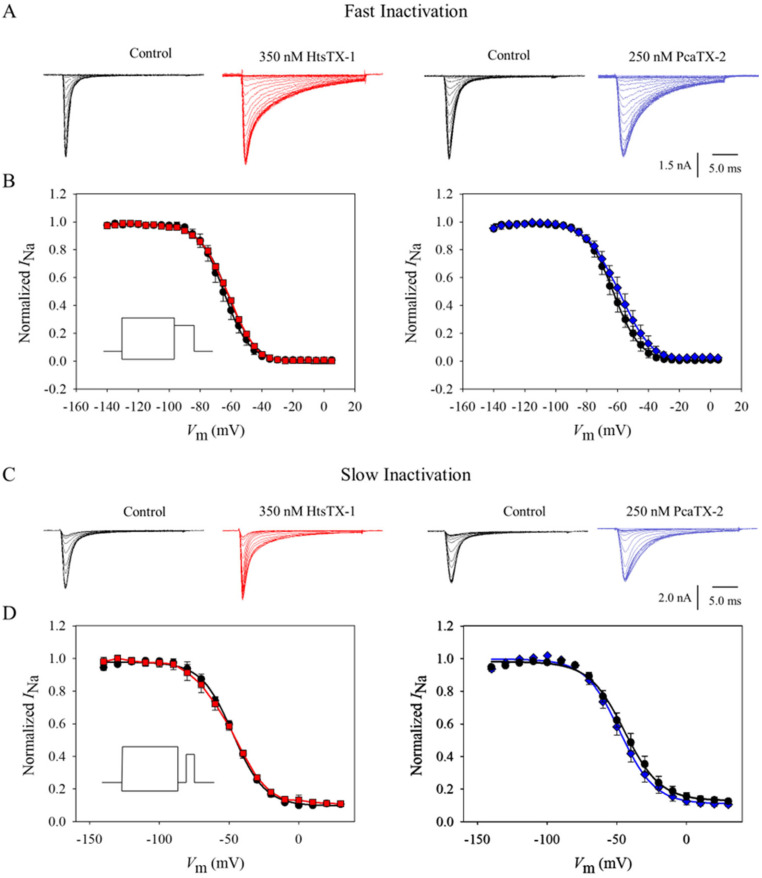
**The effect of HtsTX-1 and PcaTX-2 on Na_v_1.7 fast and slow inactivation.** (**A**) Steady-state fast inactivation (*h*_∞_) was determined using a standard two-pulse protocol. Cells were kept at a holding potential of −120 mV and subjected to 50 ms depolarizing pulses from −140 to 10 mV in +5 mV increments before (black) and after application with 350 nM HtsTX-1 (red ■) and 250 nM PcaTX-2 (blue ♦). (**B**) Currents were measured at a 20 ms depolarization step to −20 mV following the pre-pulse and plotted against the pre-pulse potential. A Boltzmann distribution was used to fit the curves. Neither the *h*_∞_ *V*_½_ nor *h*_∞_ *k* showed a statistically significant difference before (●) and after treatment of HtsTX-1 (red ■) and PcaTX-2 (blue ♦). (**C**) Slow inactivation (*SI h*_∞_) was determined using a standard three-pulse protocol. Cells were kept at a holding potential of −120 mV followed by a 10 s conditioning pre-pulse from −140 to 30 mV in +10 mV increments before (black traces) and after application with 350 nM HtsTX-1 (red ■) and 250 nM PcaTX-2 (blue ♦). (**D**) Currents were measured at a 20 ms depolarization step to −20 mV following the conditioning pre-pulse and plotted against the pre-pulse potential. A Boltzmann distribution was used to fit the curves. Neither the *SI h*_∞_ *V*_½_ nor *SI h*_∞_ *k* showed a statistically significant difference before (●) and after application with HtsTX-1 (red ■) and PcaTX-2 (blue ♦).

To further characterize the observed site 3 modulatory effects of HtsTX-1 and PcaTX-2, both steady-state fast and slow inactivation kinetics were investigated. The steady-state fast inactivation parameter (*h*_∞_)was measured using a standard two-pulse protocol. Starting at a *V*_H_ of −120 mV, conditioning pre-pulses ranging from −140 to +10 mV in 5 mV steps for 50 ms were delivered, and currents were recorded during a 20 ms depolarization step to −20 mV before (black traces) and after perfusion of HtsTX-1 (red ■) and PcaTX-2 (blue ♦). The duration of the pre-pulse potential is 50 ms, and there is no hyperpolarizing gap or return to *V*_H_ between the pre-pulse and test pulse. It has been documented that “with longer durations of pre-pulses in the steady-state fast inactivation protocol, the ratio of slow inactivated channels increases from ~5% (100 ms pre-pulse) to ~40% (2 s pre-pulse)” [52]. Representative current traces have been displayed to demonstrate their general effect on Na_v_1.7 steady-state fast inactivation (Figure 4A). Normalized sodium currents were plotted against the increase in the pre-pulse potentials and subsequently fitted with a Boltzmann distribution (Figure 4B). The voltage dependence of steady-state fast inactivation (*h*_∞_ *V*_½_) of −64.49 ± 0.85 mV before application did not significantly differ from −62.08 ± 0.67 mV after application of HtsTX-1. Similarly, the voltage dependence of *h*_∞_ V_½_ of −62.65 ± 1.27 mV before application did not significantly differ from −58.67 ± 1.32 mV after application of PcaTX-2. No statistical difference of the slope factors (*h*_∞_ *k*) was observed. Taken together, these data suggest that neither HtsTX-1 nor PcaTX-2 altered the kinetics of steady-state fast inactivation.

Modulation of the slow inactivation parameter (*SI h*_∞_) was measured using a three-pulse protocol. Cells were kept at a *V*_H_ of −120 mV followed by a 10 s conditioning pre-pulse from −140 to 30 mV in +10 mV increments before (black traces) and after perfusion of HtsTX-1 (red ■) and PcaTX-2 (blue ♦). Currents were recorded at a 20 ms depolarization step to −20 mV. Representative current traces have been displayed to demonstrate their general effect on Na_v_1.7 slow inactivation (Figure 4C). Normalized sodium currents were plotted against the increase in pre-pulse potentials and subsequently fitted with a Boltzmann distribution (Figure 4D). The voltage dependence of slow inactivation (*SI h*_∞_ *V*_½_) of −47.12 ± 0.69 mV before application did not significantly differ from −48.42 ± 0.42 mV after application with HtsTX-1. Similarly, the *SI h*_∞_ *V*_½_ of −44.43 ± 1.15 mV before application did not significantly differ from −47.86 ± 1.11 mV after application of PcaTX-2. No statistical difference in the slope factors (*SI h*_∞_ *k*) was observed. Taken together, these data suggest that neither HtsTX-1 nor PcaTX-2 altered the kinetics of slow inactivation.

### 2.4. Effect of Toxins on Na_v_1.7 Recovery from Inactivation

**Figure 5:**
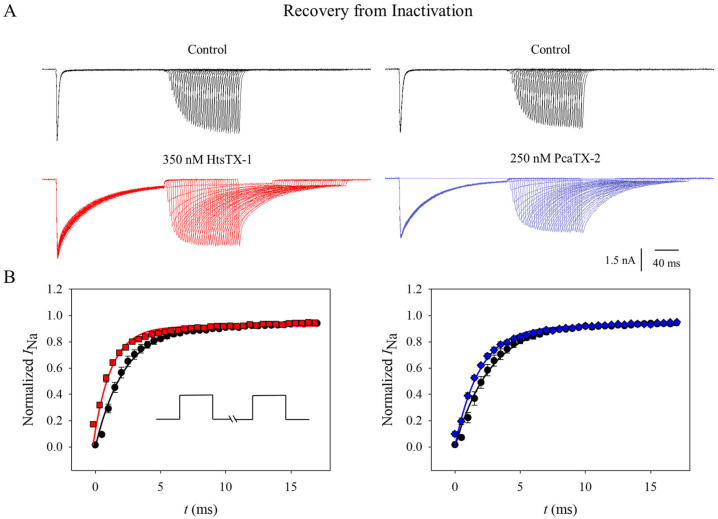
**The effect of HtsTX-1 and PcaTX-2 on Na_v_1.7 recovery of fast inactivation.** (**A**) Recovery from fast inactivation was determined using a standard three-pulse protocol. Cells were kept at a holding potential of −120 mV and subjected to a 25 ms depolarizing pulse to 0 mV followed by a series of hyperpolarizing pulses (Δ 0.5 ms) from 0 to 17 ms and then a 25 ms depolarizing pulse back to 0 mV before (black traces) and after application with 350 nM HtsTX-1 (red ■) and 250 nM PcaTX-2 (blue ♦). (**B**) Currents were recorded at a 25 ms depolarization step to 0 mV following two pre-pulses and normalized. Curves were fitted to a 2-parameter exponential. The tau (τ_R_) showed a statistically significant decrease before (●) and after application with HtsTX-1 (red ■) and PcaTX-2 (blue ♦).

An additional aspect of inactivation that was studied as an extension to voltage dependence of inactivation was the rate of recovery from inactivation using a three-pulse protocol. Cells were kept at a holding potential of −120 mV and subjected to a 25 ms depolarizing pulse to 0 mV followed by a series of hyperpolarizing pulses (Δ 0.5 ms) from 0 to 17 ms and then a 25 ms depolarizing pulse back to 0 mV before (black) and after application with 350 nM HtsTX-1 (red ■) and 250 nM PcaTX-2 (blue ♦). Representative current traces are provided to demonstrate the overall recovery from inactivation (Figure 5A). Currents measured before (black traces) and after perfusion of HtsTX-1 (red ■) and PcaTX-2 (blue ♦) at a 25 ms depolarization step to 0 mV following two pre-pulses were normalized. Curves were fitted to a 2-parameter exponential (Figure 5B). The tau (τ_R_) of recovery for the control of 2.17 ± 0.07 ms was significantly different from 1.69 ± 0.05 ms for HtsTX-1. Similarly, the τ_R_ of 2.54 ± 0.05 ms before application significantly differed from 2.11 ± 0.04 ms after application with PcaTX-2. The tau (τ) of recovery was significantly faster in the presence of the toxins. These data suggest that the channel bound to the toxin can reach a state of inactivation faster than in normal conditions. This is consistent with traditional α-scorpion toxins that putatively bind to site 3 of Na_v_1.7 and play a role in the overall delay of inactivation observed in the sodium currents.

### 2.5. Effect of Toxins on Na_v_1.7 Closed-State Inactivation

**Figure 6:**
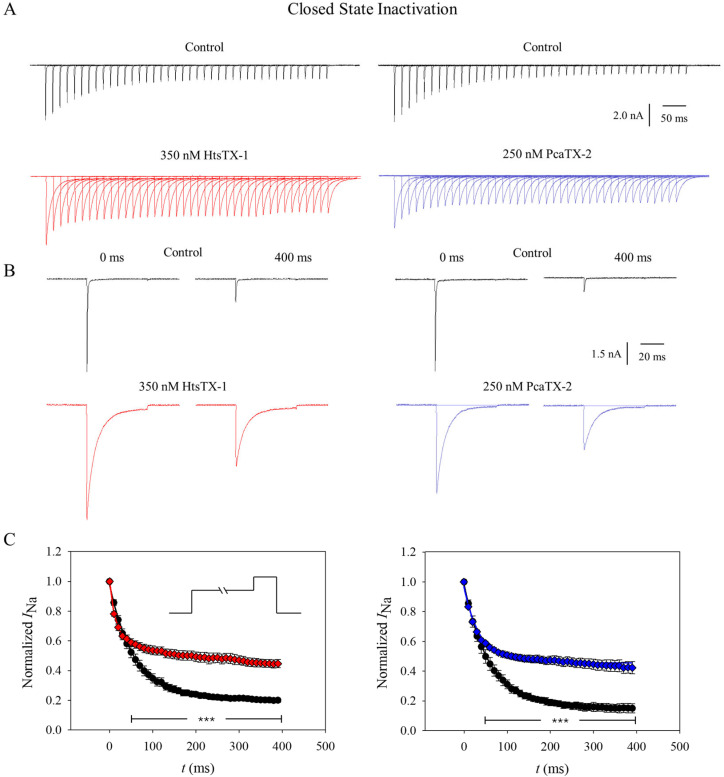
**The effect of HtsTX-1 and PcaTX-2 on Na_v_1.7 closed-state inactivation.** (**A**) Closed-state inactivation (CSI) was determined using a standard two-pulse protocol. Cells were kept at a holding potential of −120 mV and subjected to a conditioning depolarizing pulse to −60 mV from 0 to 400 ms (Δ 10 ms), followed by a 50 ms depolarizing pulse to 0 mV before (black) and after application with 350 nM HtsTX-1 (red ■) and 250 nM PcaTX-2 (blue ♦). (**B**) To further illustrate the effect of both toxins on the maximum sodium current, a single trace at 0 and 400 ms is displayed before (black traces) and after application with 350 nM HtsTX-1 (red ■) and 250 nM PcaTX-2 (blue ♦). (**C**) Currents were measured at a 50 ms depolarization step to 0 mV and fitted to a 2-parameter exponential. The tau (τ_CSI_) showed a statistically significant difference before (●) and after application of HtsTX-1 (red ■) and PcaTX-2 (blue ♦). In addition, there was a statistically significant residual of current at each time point after 70 ms in the case of HtsTX-1 and 50 ms for PcaTX-2, as shown in the *** barred region, *p* < 0.001.

To examine the effect of toxin application on the closed-state inactivation of Na_v_1.7, a standard two-pulse protocol was used. Cells were kept at a holding potential of −120 mV and subjected to a conditioning depolarizing pulse to −60 mV from 0 to 390 ms (Δ 10 ms), followed by a 50 ms depolarizing pulse to 0 mV. Representative current traces are displayed to demonstrate their general effect on Na_v_1.7 closed-state inactivation (Figure 6A). Although the inhibition of CSI is visible in the summed traces before (black) and after application with 350 nM HtsTX-1 (red ■) and 250 nM PcaTX-2 (blue ♦), a first and last trace, i.e., a depolarizing step at 0 ms and 400 ms, respectively, are provided to further emphasize the effect observed (Figure 6B).

Currents were recorded at a 50 ms depolarization step to 0 mV and fitted to a 2-parameter exponential (Figure 6C). The physiological resting membrane potential (*V*_f_) for most neurons is between −70 and −55 mV [53,54,55,56,57,58,59]. Therefore, a depolarizing pre-pulse of −60 mV was used to demonstrate a whole cell’s Na_v_1.7 contribution to a threshold stimulus. In less than 100 ms after holding at −60 mV, over ~50% of the channels are unavailable for conductance. After 350 ms, only ~20% are still available to open. After the administration of a toxin, there is a similar loss of available channels due to closed-state inactivation at the 100 ms time point. However, in contrast, there is a significantly greater number of channels still closed (available to open) at around ~45%. There was a statistically significant residual of current at every time point after 70 ms in the case of HtsTX-1 and 50 ms for PcaTX-2. In addition, the CSI tau (τ_CSI_) for the control of 57.14 ± 2.29 ms for the control was significantly different from 34.13 ± 3.30 ms for HtsTX-1. Similarly, τ_CSI_ of 58.48 ± 1.05 ms for the control was significantly different from 37.17 ± 2.78 ms for PcaTX-2. Taken together, these data suggest that in the presence of HtsTX-1 or PcaTX-2, putative site 3 modulators, the channel’s ability to experience closed-state inactivation is significantly inhibited and reduced.

## 3. Discussion

The field of toxinology has been rapidly changing from a basic science discipline focused on the identification of venom components and poisons to a pharmacology-based discipline exploring structure–activity relationships (SAR) that aid in pharmacophore developments [60]. Nowhere has this strategy been more relevant than in the understanding and treatment of neuropathies. Specifically, the study of toxins has provided the foundation for the current understanding and insight into physiologically relevant binding regions of VGSC. Although the potential of targeting Na_v_1.7 as a non-opioid alternative for pain management has yet to be realized, the demand for such an alternative has grown amidst the ongoing opioid crisis. In this pursuit, the insights gained from these toxins’ SARs are invaluable.

The first successful application of these toxin studies for pain management was the discovery of ziconotide, a peptide from the venom of the cone snail *Conus magus*. This toxin, sold under the brand name Prialt^®^, was approved by the FDA in 2004 for the “management of severe chronic pain in patients for whom intrathecal (IT) therapy is warranted and who are intolerant of or refractory to other treatment, such as systemic analgesics, adjunctive therapies or IT morphine” [61]. Although the mechanism of action is thought to be an N-type calcium channel blocker, there has been unwavering proof of concept for similar investigations [62]. More recently, the FDA approval of Journavx (Suzetrigine^®^), a small molecule selective Na_v_1.8 inhibitor, has established the effectiveness of targeting peripherally located VGSC for pain management. Whether or not analogs of Journavx could be selective for other isoforms, such as the Na_v_1.7, remains to be seen and may be unlikely. The Na_v_1.8 specific sequence, i.e., KKGS, located on the VSD of DII has been shown to be a requirement to produce its inhibitor activity. Further investigation into alternative binding mechanisms will be required to capitalize on the potential of Na_v_1.7 channels.

One feature of toxin–receptor interactions that makes these drug developments tenable is the limited number of binding sites that have been shown to elicit observable modulatory effects [32,60]. Of the seven binding sites described, site 3 and site 4 are very common for scorpion and spider toxins. Site 3, located on the S3-S4 Linker of DIV, has two prominent effects on VGSCs: an observed slowing of the τ of inactivation and an increase in the overall sodium conductance [38,63,64]. In contrast, site 4, associated with the S3-S4 Linker of DII, shifts the voltage dependence of activation and alters sodium conductance [40,65]. Unfortunately, as more toxins have been described, a greater understanding of these toxins’ interaction is needed to explain the differences seen in modulation, e.g., potency, selectivity, and promiscuous activity. The complementarity of protein–protein interactions (PPI) may hold the information needed for development to become truly pathology/patient specific.

Currently, the VGSC’s modulation of both activation and inactivation is still an underrealized and viable target for drug development. Therefore, in this investigation, a focus on site 3 modulation of inactivation was examined. Two novel toxins, i.e., HtsTX-1 from the Order Scorpiones and PcaTX-2 from the order Araneae, were purified and characterized. Both toxins demonstrated activity consistent with site 3 modulation, demonstrated prolongation of inactivation, increased peak sodium current, and an enhancement of the recovery from fast inactivation [32,33,35,36,37]. Although there has been a distinct divergence between these two orders, dating back 325–375 million years ago, the preservation of ion channel modulation is evident [66]. More specifically, a similar structural motif for ion channel modulators is becoming clearer. The three cysteine-bridged knottins, although differing in amino acid sequence, have similar characteristics that may help to further clarify the site 3 binding and subsequent effects on channel inactivation. Neither toxin has a large effect on the voltage dependence on activation. Although PcaTX-2 does display a small but significant shift in conductance, it is much smaller than PcaTX-1, as described previously [45]. It was concluded that this is due to a concomitant binding to site 4.

The sequence of HtsTX-1 did not conform with the typical profile for α-scorpion toxins, as they are typically 60–70 residues and contain four Cys bridges. By contrast, HtsTX-1 has 32 residues and three Cys bridges and is smaller than the typical α-scorpion toxin. In terms of sequence homology, the sequence for HtsTX-1 was run against the UniProtKB reference proteomes + Swiss prot database. Of the nine possible library matches, only one was a 32-residue peptide identified as Peptide II.10.10 (Entry C0HJW4) from the scorpion *Centruroides tecomanus* with 60.7% homology. This peptide was identified in a study where several potassium channel inhibitors were characterized, but it did not display any effect on voltage-gated sodium channels [67]. Since this peptide does not have a known mechanism of action, it is difficult to make assumptions about the relevance of this shared homology.

The sequence of PcaTX-2 showed that it is a 33-residue peptide with three Cys bridges. HtsTX-1 did not share significant sequence homology or relevant bridging motifs despite sharing the same number of bridges. Looking at other toxins from the same genus, PcaTX-2 shared ~35% homology in sequence with PcaTX-1, and both have the same Cys bridge configuration as well as an amidated C-terminus. PcaTX-2 shares ~58–73% homology compared to toxins from other theraphosids (tarantulas) archived in UniProt. Like PcaTX-1, all similar toxins share the Cys bridge motif, i.e., knottins, and an amidated C-terminus. However, unlike other similar toxins, PcaTX-2 showed the smallest shift in voltage-gated kinetics of activation. HtsTX-1 showed no significant shift at all. Taken together, this suggests that HtsTX-1 and PcaTX-2 have less binding affinity or potency at site 4 but still demonstrate potent modulation at site 3. This will allow for future studies that focus on site 3 modulation to better elucidate the effect on inactivation.

Consistent with the findings of previously described site 3 modulators, both HtsTX-1 and PcaTX-2 demonstrated the characteristic delay in inactivation. Despite this effect, neither toxin caused a significant change in the kinetics of steady-state fast inactivation nor slow inactivation. While HtsTX-1 did not change the conductance kinetics, PcaTX-2 showed a small depolarizing shift to the conductance kinetics, which suggests that it is acting promiscuously at a separate binding region, i.e., site 4. With a deeper look at the rate of recovery from inactivation, both toxins showed a significant difference in the channel’s ability to recover from inactivation, suggesting that the toxins destabilize the inactivation state and the channel can be closed for reactivation more readily.

One remaining effect that has been understudied and may further help characterize the PPI of site 3 modulation is the phenomenon of CSI. Due to the subtle differences in the S4 transmembrane segments, each domain responds to charges in a differential manner. As a result, each VSD has independent movements and a channel can transition from a primed or closed state to a refractory or inactivated state; i.e., CSI is the activation of DIII-DIV VSD prior to other domains, causing inactivation before the channel opens. Both toxins in this investigation alter the channel’s ability to transition to an inactivated state at physiologically relevant membrane potentials. This is demonstrated by the channel’s hinderance to undergo normal CSI. While changes to physiological CSI in VGSCs with gene mutations have been characterized, e.g., Brugada Syndrome, modifications due to xenobiotic interactions have garnered far less attention. A better understanding of toxin SAR regarding CSI will help to establish a deeper understanding of this physiologically relevant phenomenon. Ultimately the enhancement of CSI could dampen excitability, which may prove to be an effective therapeutic alternative to producing analgesia.

## 4. Conclusions

In this manuscript, we have characterized two novel toxins from two distinct orders that modulate site 3 in the VGSC Na_v_1.7 in a parallel investigation. Neither significantly alters the voltage dependence of steady-state fast or slow inactivation, but both significantly alter the channel’s recovery from inactivation as well as the closed-state inactivation. This approach allows for a more complete clarification of site 3 PPI, and more importantly, demonstrates the value of investigations of toxins that alter a phenomenon that may be exploited for many pathologies that are a result of overexcitable sodium channels. Closed-state inactivation has the potential of keeping pain-sensing neurons in a refractory state at physiological membrane potential. If a clearer picture of the SAR of site 3 toxins is established, molecular development that is more specific, potent, and use-dependent may be possible.

## 5. Materials and Methods

### 5.1. Reagents and Chemicals

All reagents utilized were purchased from Avantor Sciences (Radnor, PA, USA) or Sigma-Aldrich (Saint Louis, MO, USA) as ACS reagent grade or better; LC/MS grade acetonitrile was used for high resolution mass spectrometric determinations.

### 5.2. Venom Collection and Preparation

Specimens of *H. junceus* and *P. regalis* were housed as previously described [45]. The only addition was the potential field applied to the dorsal base of the telson of *H. junceus*.

Venoms were freeze-dried and preserved as previously described [45].

### 5.3. Reversed-Phase Chromatography (RPC) Purifications

Toxins were separated as previously described [45].

### 5.4. Reversed-Phase Chromatography (RPC) Determinations

All reversed-phase chromatography fractions were suspended into the initial mobile phase buffer and injected into a Waters Acquity^®^ (Wexford, Ireland) Ultra Performance Liquid Chromatograph (UPLC) equipped with an Acquity UPLC BEH C18 1.7 μm 2.15 × 50 mm column at a flow rate of 0.50 mL/min, a 1.00 min hold at 90:10 A:B (mobile phase A = 0.10% formic acid; mobile phase B = acetonitrile) followed by a binary mobile phase gradient to 60:40 A:B in 40.00 min, then 15:85 A:B in 2.00 min, lastly held for 7.00 min providing proper peak shape, separation, and reduction in interferences.

### 5.5. Toxin Digestions and Modifications

Toxins were digested as previously described [45].

All protein digestions were performed with slight variations to protocols provided by Thermo Scientific [68].

### 5.6. Accurate Mass Determinations

To determine a toxin’s monoisotopic mass, aliquots were analyzed by a Waters UPLC Acquity^®^ (Wexford, Ireland) interfaced with a Synapt^®^ (Manchester, UK) G2-Si HRMS equipped with an ESCI^®^ (Manchester, UK) z-spray atmospheric pressure ionization source. Lock mass correction using [Glu1]-fibrinopeptide B human as a reference was used before processing.

High resolution mass spectra were acquired as previously described [45].

### 5.7. Cell Culture

Catecholamine A-differentiated (CAD) cells were maintained as previously described [69].

### 5.8. Whole Cell Recording

Whole-cell voltage clamp recordings were performed as previously described [69] with the following changes: HEKA PatchMaster V 2 × 90.5 and a holding potential (*V*_H_) of −120 mV. Sodium currents were recorded with an internal solution that contained (in mM) the following: 110 CsF, 10 NaCl, 10 CsCl, 10 EGTA, and 10 HEPES (pH 7.2 with CsOH) and an external solution of the following: 100 NaCl, 20 tetraethylammonium (TEA) chloride, 2 CaCl_2_, 1 MgCl_2_, 10 D-Glucose, and 10 HEPES (pH 7.3 with NaOH). In addition, slow inactivation was determined using a standard two-pulse protocol. At a holding potential of −120 mV, conditioning pre-pulses of 10 s durations ranging from −140 to 30 mV in 10 mV steps were delivered, and currents were measured at a depolarization step to −20 mV (20 ms). Recovery from fast inactivation was measured at 0 mV for 25 ms following increasing recovery periods (0–17 ms) and a pre-pulse at 0 mV for 25 ms. Closed-state inactivation currents are elicited by a 50 ms depolarization step to 0 mV following a step at −60 mV for a duration of 0–400 ms, increasing 10 ms for each step.

### 5.9. Data Analysis and Statistics

Mass spectra and electrophysiological traces were processed as previously described [45] with the following additions.

The time constant (τ_F_) for fast inactivation was determined from an exponential curve and computed in two steps using the HEKA FitMaster V 2 × 90.5: (1) semi-logarithmic regression of x vs. ln (y) and (2) abs (1/slope).

Slow inactivation curves were fitted with a single-phase Boltzmann function where *V* is the test potential, *V*_1/2_ is the potential at which 50% of the maximal conductance is activated, *k* is the slope factor as follows:

IIMax = 11 + e(V−V12)/k

Recovery from steady-state fast inactivation was fitted with an exponential function, A is the amplitude, t is time and τ is the time constant as follows:

$$\frac{I}{I_{Max}}\;=\;A(1\;-\;e^{-\frac{t}{\tau }})$$

Closed-state inactivation was fitted with an exponential function, A is the amplitude, *t* is time and τ is the time constant as follows:

$$\frac{I}{I_{Max}}\;=\;C\;+\;A(e^{-\frac{t}{\tau }})$$

## Figures and Tables

**Table 1:** Effects of HtsTX-1 and PcaTX-2 on Na_v_1.7 parameters.

Parameter	Control	HtsTX-1	Control	PcaTX-2
*I V*_½_ (mV)	−32.42 ± 1.11	−35.13 ± 1.21	−33.04 ± 0.95	−30.43 ± 0.86
*I V*_rev_ (mV)	61.1 ± 0.76	59.1 ± 0.62	57.6 ± 1.08	60.7 ± 1.19
*G V*_½_ (mV)	−26.75 ± 0.29	−29.35 ± 0.36 ***	−27.35 ± 0.29	−21.62 ± 0.30 ***
*G k*	3.41 ± 0.25	3.61 ± 0.31	3.45 ± 0.25	5.05 ± 0.25 **
*h*_∞_ *V*_½_ (mV)	−64.49 ± 0.85	−62.08 ± 0.67	−62.65 ± 1.27	−58.67 ± 1.32
*h_∞_* *k*	−8.19 ± 0.31	−9.06 ± 0.26	−8.61 ± 0.39	−10.16 ± 0.57
*SI h*_∞_ *V*_½_ (mV)	−47.12 ± 0.69	−48.42 ± 0.42	−44.43 ± 1.15	−47.86 ± 1.11
*SI h_∞_ k*	−11.38 ± 0.61	−12.93 ± −0.94	−12.81 ± 1.03	−12.49 ± 1.00
*τ*_R_ (ms)	2.17 ± 0.07	1.69 ± 0.05 ***	2.54 ± 0.05	2.11 ± 0.04 ***
*τ*_CSI_ (ms)	57.14 ± 2.29	34.13 ± 3.30 ***	58.48 ± 1.05	37.17 ± 2.78 ***

*V*_½_, half-maximal potential of *I*_Na_; *V*_rev_, reversal potential; *G*, Na^+^ conductance; *h*_∞_ steady-state inactivation parameter; *V*_½_ and *k*, mid-activation, or inactivation voltage and slope factor of the fitted *G/V* and *h*_∞_ curves; *SI* denotes parameters related to slow inactivation; *τ*_R_, time constant of the recovery from steady-state fast inactivation; *τ*_CSI_, time constant of closed-state inactivation; unpaired *t*-test, ** *p* < 0.01, *** *p* < 0.001; values are presented as mean ± SEM (*n* = 5).

## Data Availability

The original contributions presented in this study are included in the article. Further inquiries can be directed to the corresponding author.

## Abbreviations

The following abbreviations are used in this manuscript:

Da	Dalton
OSI	Open-state Inactivation
CSI	Closed-state Inactivation
HRMS	High-resolution Mass Spectrometry
m/z	mass to charge ratio
*t*_R_	Retention Time
ToF	Time of Flight
TIC	Total Ion Chromatogram
UPLC	Ultra-performance Liquid Chromatography
VSD	Voltage-gated Domain
VGSC	Voltage-gated Sodium Channel
PPI	Protein–Protein Interaction
HtsTX-1	Heteroctenustoxin-1
PcaTX-2	Poecilotheriatoxin-2
